# In Vitro Microbial Adhesion on the Surfaces of Various Polytetrafluoroethylene Membranes Used in Guided Bone Regeneration

**DOI:** 10.3390/dj13070301

**Published:** 2025-07-02

**Authors:** Adel Al-Asfour, Maria G. Katsikogianni, Maribasappa Karched, Syed Saad Bin Qasim, Branko Trajkovski, Gregor-Georg Zafiropoulos

**Affiliations:** 1 Department of Surgical Sciences, College of Dentistry, Kuwait University, Safat 13110, Kuwait; adel.alasfour@ku.edu.kw (A.A.-A.); ggzafi@gmx.de (G.-G.Z.); 2 School of Chemistry, Faculty of Life Sciences, University of Bradford, Bradford BD7 1DP, UK; m.katsikogianni@bradford.ac.uk; 3 Oral Microbiology Research Laboratory, Department of Bioclinical Sciences, College of Dentistry, Kuwait University, Safat 13110, Kuwait; 4 Dental Materials Research Laboratory, Department of Bioclinical Sciences, College of Dentistry, Kuwait University, Safat 13110, Kuwait; sayed.binqasim@ku.edu.kw; 5 Institute of Molecular Biology, Bulgarian Academy of Sciences, 1113 Sofia, Bulgaria; biobtanko@gmail.com

**Keywords:** bone regeneration (guided)/GBR, PTFE, membranes, surface free energy, microbial attachment, *P. gingivalis*, *S. mutans*, *C. albicans*

## Abstract

**Aim:** The aim of this study was to evaluate the adhesion of oral microorganisms on the surfaces of polytetrafluoroethylene (PTFE) membranes used in guided bone regeneration (GBR) procedures. **Materials and Methods:** In this study, three oral microorganisms (*Streptococcus mutans*, *Porphyromonas gingivalis*, and *Candida albicans*) were used, and six PTFE membranes were characterized by their surface roughness, contact angle (CA), and surface free energy (SFE). Microbial hydrophobicity was investigated, and adhesion was examined via DNA extraction and quantitative real-time PCR. **Results:** Significant differences were noted amongst the membranes with respect to SFE, CA, and roughness (*p* < 0.001). *S. mutans* was the most hydrophobic microorganism, followed by *C. albicans* and *P. gingivalis*. SEM analyses confirmed that the microorganisms adhered to all membranes, with Surgitime being the membrane that attracted the highest number of *S. mutans* (*p* < 0.001) and *P. gingivalis* (*p* < 0.001). By contrast, OsseoGuard-TXT was one of the membranes that attracted the lowest number (*p* < 0.001) of all three tested species. **Conclusions:** The results showed that microbial adhesion to PTFE membranes was affected by the membrane surface roughness and SFE, as well as the characteristics of the microorganisms. The most hydrophilic bacteria adhered the least to all the tested membranes, whereas membranes with a low surface roughness and high SFE attracted the lowest number of all the tested microbes. These results may guide the selection of an appropriate GBR membrane.

## 1. Introduction

Guided bone regeneration (GBR) is a treatment modality applied in implant and oral surgery procedures, leading to the regeneration of bone defects [1]. Membranes are used as barriers to cover bone defects, prohibiting the migration of non-osteogenic tissues into the defect [1]. Non-resorbable membranes are mostly used in GBR surgeries and comprise either expanded (e-) or dense (d-) polytetrafluoroethylene (PTFE). In cases of exposure during the healing period, membranes may be colonized by oral microorganisms, increasing the potential risk of infection of the surrounded tissues, as well as the covered bone defects, thus compromising the treatment [1,2,3]. Factors influencing microbial adhesion to a biomaterial surface include the physical properties of the materials, such as the surface free energy (SFE) and roughness, and microbial hydrophobicity, amongst others [4,5].

The surface free energy (SFE) critically determines the wettability of solids by liquids [6] and can be calculated from measurements of the contact angle (CA) using Young’s equation [7]. The factors that play a role in influencing the accuracy of CA measurements are not limited to surface rigidity; they also include surface roughness, the physical and chemical properties of a surface, the type of measured liquid, and humidity [8]. The roughness of the substratum, i.e., the membrane surface, is considered a key determinant in microbial adhesion, as it is dependent on the surface topography and stiffness [4,8,9].

While existing studies have established the role of surface roughness and texture in microbial adhesion to PTFE membranes, the combined influence of the surface free energy, microbial hydrophobicity, and membrane surface roughness on the adhesion of key oral pathogens has not been studied to the same extent. In previous work, we demonstrated the adhesion of an opportunistic oral pathogen, *Candida albicans*, to PTFE membranes and how it is influenced by their surface texture and roughness [10]. In this study, we extended that work to investigate the adhesion of additional oral microorganisms, *Streptococcus mutans* and *Porphyromonas gingivalis*, alongside *C. albicans*, with a focus on how their adhesion is influenced by the SFE of the microorganisms and membranes, the surface roughness of the membranes, and the hydrophobicity of the microorganisms examined.

## 2. Materials and Methods

### 2.1. Membrane Specifications

In this study, we evaluated six standard non-titanium-reinforced PTFE membranes, focusing exclusively on their soft tissue contact surfaces (Table 1).

### 2.2. Microbial Species and Culture Conditions

Two bacterial strains and one yeast strain were cultured and tested. More specifically, *Streptococcus mutans* CCUG 11877 and *Porphyromonas gingivalis* ATCC 33277 were cultured on brucella blood agar containing 5% sheep blood; they were then incubated in 5% CO_2_ and in anaerobic conditions, respectively, for 2–3 days. The yeast strain *Candida albicans* ATCC 24433 was aerobically cultured on *Sabouraud* dextrose agar (SDA; BD, Franklin Lakes, NJ, USA) at 37° C for 24 h. All cultures were observed under a stereo microscope to confirm their colony morphology and to check for any contamination prior to using them in experiments.

### 2.3. Material Surface Roughness

The stationary surface roughness was quantitatively evaluated using an optical profilometer (LEICA DCM 8, Leica Microsystems CMS GmbH, Wetzlar, Germany), following an established methodology [11]. Measurements were performed with a 10× objective lens under ambient conditions, requiring no sample preparation. Prior to imaging, specimens were positioned on a clean glass slide. In accordance with ISO25178 (ISO 2012) [12], the aerial surface roughness parameters—specifically the arithmetic average height (RA; Robust Gaussian filter 0.25 mm) and root mean square gradient (Rq)—were measured across a 500 µm sampling length. Three readings were taken from the surface, expressed as µm. The structural differences between Cytoplast™ TXT-200 and OsseoGuard^®^-TXT membranes necessitated distinct measurement approaches. Thus, for these membranes, surface characterization data were obtained by separately analyzing points from both the hexagonal indentations and intervening non-textured regions.

### 2.4. Measurement of Contact Angles and Surface Free Energy Calculations for Materials and Microorganisms

A drop of liquid (3 μL), distilled water, as a polar liquid, and diiodomethane (DII; Sigma-Aldrich Chemie GmbH, Taufkirchen, Germany), as a non-polar liquid, were added to the surface of the membrane with a syringe at 20 °C. The contact angle (CA) was measured via the sessile drop method using a drop shape analyzer (DSA100B; Krüss, Hamburg, Germany) and determined using a drop shape analysis program (ADVANCE 1.7.2.1; Krüss, Hamburg, Germany). To minimize experimental errors, 10 readings were performed for each specimen, and their average value was reported. For determining the dynamics of CA changes with time, the shape of the liquid droplet was recorded using a camera (with a resolution of 0.01° and accuracy of 0.1°), starting from zero seconds and lasting 5 min.

For the microbial cell measurements, the procedure followed the methodology established by Busscher [13], involving microbial layers deposited on membrane filters. Specifically, a 20 mL suspension of each microbial strain in phosphate-buffered saline (PBS)—prepared after washing—was filtered through a 0.2 μm membrane filter under negative pressure. This filtration process created a dense bacterial lawn on the filter surface, which was subsequently air-dried for a period of three hours to achieve a ‘dried-plateau’ state. At this critical point, when the surface moisture between the cells had fully evaporated, the microbial cells themselves remained adequately hydrated. This optimal drying condition enabled the precise measurement of contact angles for each probe liquid used in the experiments, which were performed according to the methods of Owens and Wendt, as well as Rabel and Kaelble (Supplementary Materials) [14].

### 2.5. Surface Hydrophobicity of the Microorganisms

The microbial species were suspended in sterile PBS [OD_600_ = 1 (A0)] and added to an equal volume of n-Hexadecane (Sigma Chemical Co., St. Louis, MO, USA). The mixture was vigorously vortexed for 1 min at the maximum setting. Then, the upper aqueous phase was gently pipetted into a new microcentrifuge tube, and the OD_600_ was measured (A1) [15]. The percent hydrophobicity was calculated as follows:

(1) $$\%\;\mathrm{Hydrophobicity}=\frac{A0-A1}{A0}\times 100$$

### 2.6. Microbial Adhesion on Various Membranes

The membranes were cut into 10 mm circular disks and placed in 24-well cell culture plates, with each well containing 900 μL of brucella broth (BD, Franklin Lakes, NJ, USA). Then, 100 μL of a standardized concentration (A0) of the microorganisms was added to each well, excluding the control, and incubated for 2 days using the culture conditions described above. Subsequently, the membranes were transferred to a new plate, washed thrice in PBS, transferred into sterile microcentrifuge tubes containing 150 μL of nuclease-free water, and vortexed for 1 min. Subsequently, the suspensions were centrifuged at 10,000× *g* for 5 min, and the resulting pellets containing intact microbial cells were subjected to DNA purification.

For examination under a scanning electron microscope (SEM) (JSM IT 200; JEOL, Akishima, Japan), a set of microbial-treated membrane disks were fixed in 3% glutaraldehyde in PBS for 2 h on a rotator, followed by overnight refrigeration. After three PBS washes, they were treated with 1% osmium tetroxide for 2 h, dehydrated in acetone gradients (30–100%) on a rotator for 10 min each, and dried in a critical point dryer. The samples were then mounted on aluminum stubs with carbon double-adhesive tape and gold-coated on a DII-29030SCTR Smart Coater for 4 min. Then, the specimens were observed, and images of the samples were captured using a Jeol IT 200 Scanning Electron Microscope (JEOL Ltd., Akishima, Tokyo, Japan) with an accelerating voltage of 20 KV.

### 2.7. Characterization of Microbial Adhesion Using DNA Extraction and Purification

DNA from the three reference microbial strains and from the membrane-detached microbial cells was purified using a DNeasy DNA Purification Kit (Qiagen GmbH, Hilden, Germany). An enzymatic lysis buffer containing Tris EDTA buffer (20 mM Tris, 2mM EDTA) with 1.2% Triton X-100 and lysozyme was used (St. Louis, MO, USA), and purified DNA was eluted in nuclease-free water, with concentrations measured via the UV spectrophotometry method using NanoDrop^TM^ 1000 (Thermofisher, Waltham, MA, USA).

### 2.8. Quantitative Real-Time PCR (qPCR)

For qPCR quantification of the microbial species adhered to the membranes, previously validated species-specific primers for the 16S rRNA gene were used (Supplementary Materials) [16,17]. Subsequently, a reaction mixture for qPCR—10 µL of the SYBR Green master mix (Power SYBR Green Kit; Applied Biosystems, Waltham, MA, USA), 0.5 µL each of forward and reverse primer (0.2 µM), 7 µL of nuclease-free water, and 2 µL of the DNA template—was prepared. The following temperature profile was used for amplification on an ABI 7500 Fast RT-PCR machine (Applied Biosystems, Waltham, MA, USA): after 10 min of initial denaturation at 95 °C, 40 cycles lasting 15 s at 95 °C, 30 s at 52–56 °C (depending on the primer pair), and 30 s at 72 °C were run. The elongation step was utilized for fluorescent signal acquisition, and the software SDS 1.4.0v (Applied Biosystems, Waltham, MA, USA) was used to analyze the data. The process used serial dilutions of DNA from the aforementioned species, and cycle threshold (Ct) values were plotted against the microbial cell content deduced (cells/mL) for each species to create standard curves using the software mentioned above. For establishing standard curves, the microorganisms’ counts were determined using the RT-PCR software SDS 1.4.0v mentioned above.

### 2.9. Gibbs Free Energy Change upon Microbial Adhesion

The tendency of adhesion is expressed by the Gibbs free energy change in the process according to the equation $\Delta G_{d0}^{adh}=\gamma_{BS}-\gamma_{BL}-\gamma_{SL}$, where $\Delta G_{d0}^{adh}$ (J/m^2^) is the free energy of adhesion per unit area of a bacterium to a substratum surface in a suspending liquid, when the separation distance (*d*) between the bacterium and the surface tends to zero and the dispersion–polar approach of the thermodynamic theory is used (Supplementary Materials) [18,19].

### 2.10. Statistical Analysis

All tests were conducted in triplicate, with each repeated three times, yielding consistent results. Subsequently, the mean values were used for analysis. The microbial quantities (cells) were log-transformed after adding one to all data to handle zeroes in the statistical analyses. The membrane property data were used without log transformation, and the normality of the data was tested via Skewness and Kurtosis values, Shapiro–Wilk *p* values, and histograms. Nonparametric Kruskal–Wallis one-way ANOVA or Mann–Whitney U tests were used to compare the membranes, given the fact that the data were not normally distributed. Pearson correlation analysis was used to assess the association between membrane properties and microbial adhesion, and the significance level for the rejection of the null hypothesis was set at α = 0.05. Statistical analyses of the data were conducted using IBM SPSS Statistics software (v. 28; Armonk, NY, USA).

## 3. Results

### 3.1. Roughness, CAs, and SFE of Membranes and Microorganisms

Data reflecting various characteristics of the PTFE membranes and the three microbial strains examined are presented in Table 2.

**Figure 1:**
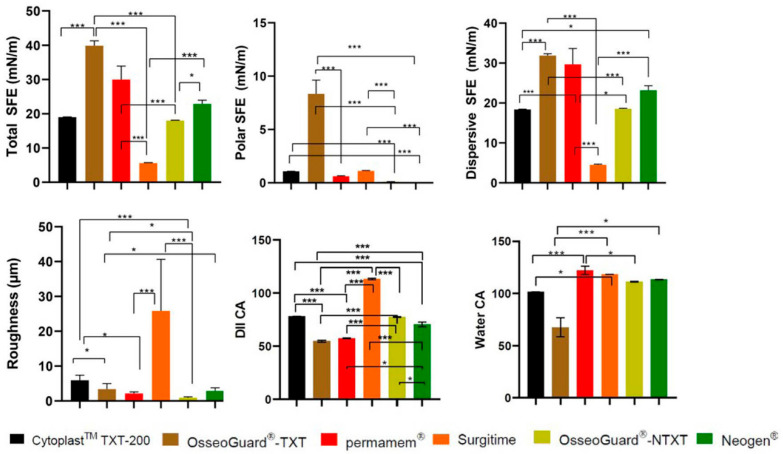
A comparison of roughness and contact angles of the PTFE membranes examined. CA: contact angle; DII: diiodomethane; *: *p* < 0.05; and ***: *p* < 0.001.

Cytoplast^TM^ TXT-200 exhibited moderate roughness (RA), with relatively low hydrophilicity, and OsseoGuard^®^-TXT had lower RA and reduced hydrophilicity. Permamem^®^ was characterized by low RA and high hydrophobicity, and Surgitime featured a notably rough surface with a large CA, signifying considerable hydrophobicity. OsseoGuard^®^-NTXT showed the lowest RA with a large CA, indicating strong hydrophobicity. NeoGen^®^ was found to have moderate RA and hydrophobicity (Figure 1).

The DII CA and SFE findings suggest that (A) Cytoplast^TM^ TXT-200 had a balanced surface, with contributions from both polar and dispersive components and a relatively low total SFE with dominant dispersive SFE; (B) OsseoGuard^®^-TXT had a relatively low-polarity surface and relatively elevated total SFE; (C) permamem^®^ showed low-polarity characteristics, whereas its total SFE indicated a moderate surface; (D) in the case of Surgitime, a predominantly non-polar surface was indicated with a low total SFE; (E) OsseoGuard^®^-NTXT had a balanced surface with both polar and dispersive properties and a moderate total SFE; and (F) NeoGen^®^ was found to have an extremely low polar SFE (Figure 1).

Among all the tested membranes, OsseoGuard^®^ TXT was found to have the greatest polar SFE, and Surgitime was found to have the greatest roughness (Figure 1).

The CA and SFE measurements obtained indicate that *S. mutans* had the highest water CA and the lowest polar SFE, therefore being the most hydrophobic of the three strains. Compared to *C. albicans*, *P.* gingivalis was found to have a lower water CA and slightly higher total SFE, indicating that *P. gingivalis* is slightly more hydrophilic than *C. albicans*.

### 3.2. Hydrophobicity of Microbial Species

*P. gingivalis* exhibited a lower mean hydrophobicity (57.4%) than *C. albicans* (77.2%) and *S. mutans* (83%), and all three microorganisms had hydrophobicity percentages above 50%, with *S. mutans* being the most hydrophobic (83%), followed by *C. albicans* (77.2%) and *P. gingivalis* (57.4%).

### 3.3. RT-PCR Quantification of the Microorganisms Adhering to the Membranes

**Figure 2:**
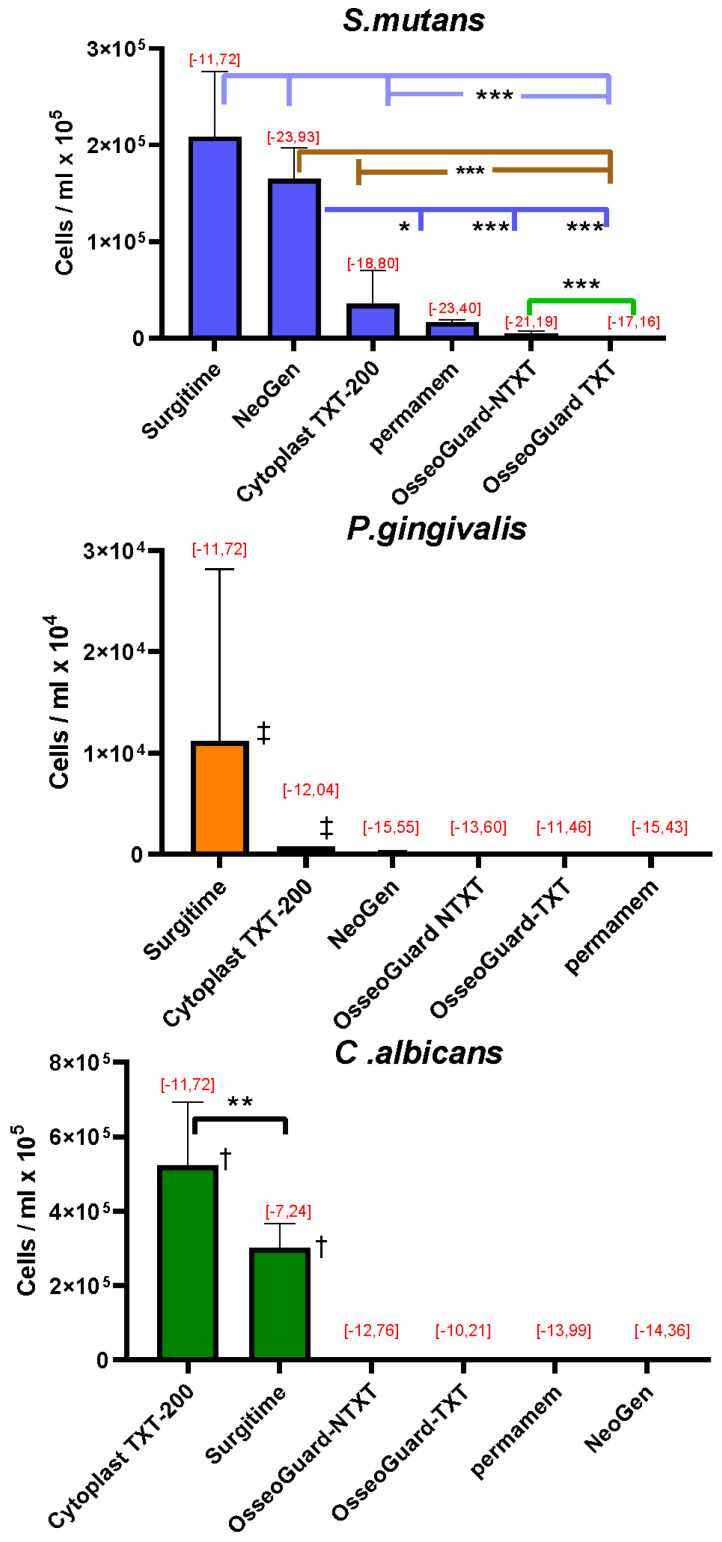
The number of microorganisms adhering to the membranes (mean ± SD) and Gibbs free energy change calculations [$\Delta G_{adh}^{d-p}$, mJ/m^2^], with values indicated in red. Membranes are listed according to decreasing numbers of the adhered microorganisms. Significant differences: * = *p* < 0.05; ** = *p* < 0.01; *** = *p* < 0.001; and ‡ = *p* < 0.001 compared to all other membranes; † = *p* < 0.001 between all other membranes (exception: the comparison between Cytoplast^TM^ TXT-200 and Surgitime for *C. albicans*).

Quantitative RT-PCR analysis of the microorganisms grown on the membranes revealed that high concentrations of *S. mutans* adhered to Surgitime and NeoGen^®^, followed by concentrations that adhered to Cytoplast^TM^ 200-TXT, permamem^®^, OsseoGuard^®^-NTXT, and OsseoGuard^®^-TXT (Figure 2). For *P. gingivalis*, the greatest concentration of cells adhered to Surgitime, followed by Cytoplast^TM^ 200-TXT. Only minimal cell adhesion of *P. gingivalis* was observed on NeoGen^®^, OsseoGuard^®^-TXT, OsseoGuard^®^-NTXT, and permamem^®^. *C. albicans* cells showed the greatest adherence to Cytoplast^TM^ TXT-200, followed by Surgitime. Relatively low *C. albicans* cell numbers per ml were observed on OsseoGuard^®^-NTXT and OsseoGuard^®^-TXT, with cell adhesion being very low on permamem^®^ and minimal on NeoGen^®^.

The Gibbs free energy changes ($\Delta G_{adh}^{d-p}$) upon adhesion of all three microbial species tested had negative values (Figure 2), indicating that microbial adhesion was favorable on all surfaces [18].

### 3.4. Microbial Adhesion to the Membranes Examined via SEM

**Figure 3:**
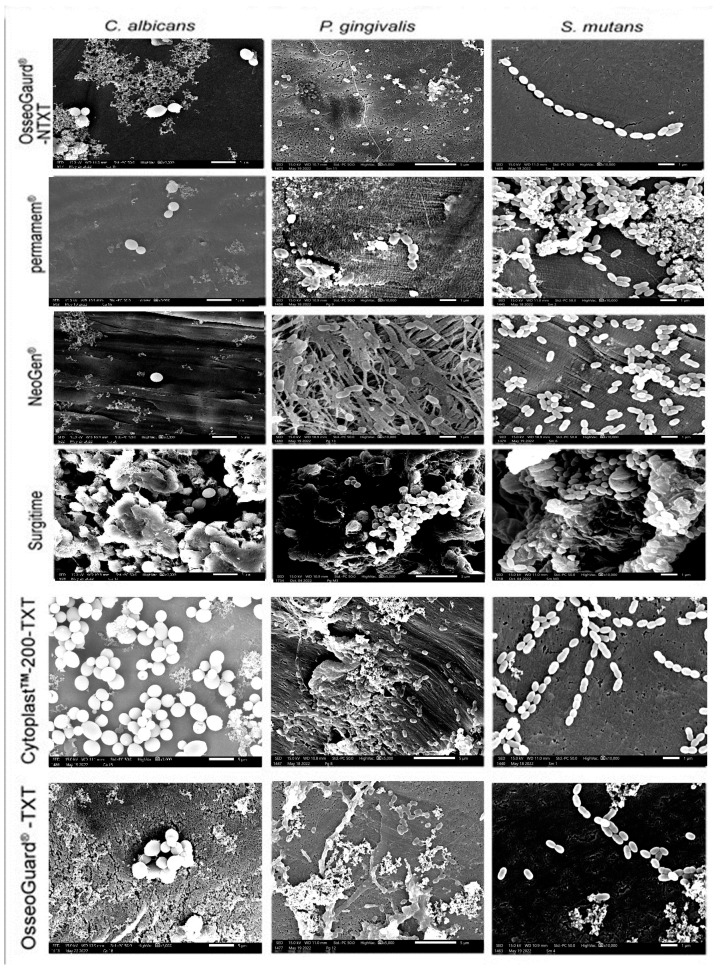
Representative SEM images of microbial adhesion to the various membranes. Bar = 1 µm and magnification = 10,000×. Images for *C. albicans* were reproduced from Asfour et al. (2024) [10] under the open access Creative Commons CC BY 4.0 license.

A thorough visual inspection revealed that microorganism adhesion to PTFE membranes varied across membrane types and microbial species, with all three microorganisms growing on the membranes as microcolonies (Figure 3). In addition to patchy and scattered adhesion, small clusters of microorganism cells were seen. On permamem^®^, *S. mutans* adhered—but also grew—as a biofilm mass, whereas *P. gingivalis* and *C. albicans* only attached as clusters of cells. Interestingly, tiny microbial clumps and free individual cells sparsely adhered to the textured membranes Cytoplast^TM^ TXT-200 and OsseoGuard^®^-TXT, as well as to the nontextured membranes OsseoGuard^®^-NTXT and NeoGen^®^, in the absence of biofilm formation evidence. Surgitime was the only membrane to which all three microorganisms adhered and formed thick biofilms. No differences in microbial adhesion were seen between OsseoGuard^®^-TXT and OsseoGuard^®^-NTXT (Figure 3).

### 3.5. Correlations Between Microbial Adhesion, Membrane Roughness, and SFE

For both textured and non-textured membranes, microbial adhesion correlated directly (*p* < 0.01) with roughness and CA (r = 0.640, r = 0.496 for water and DII, respectively).

**Figure 4:**
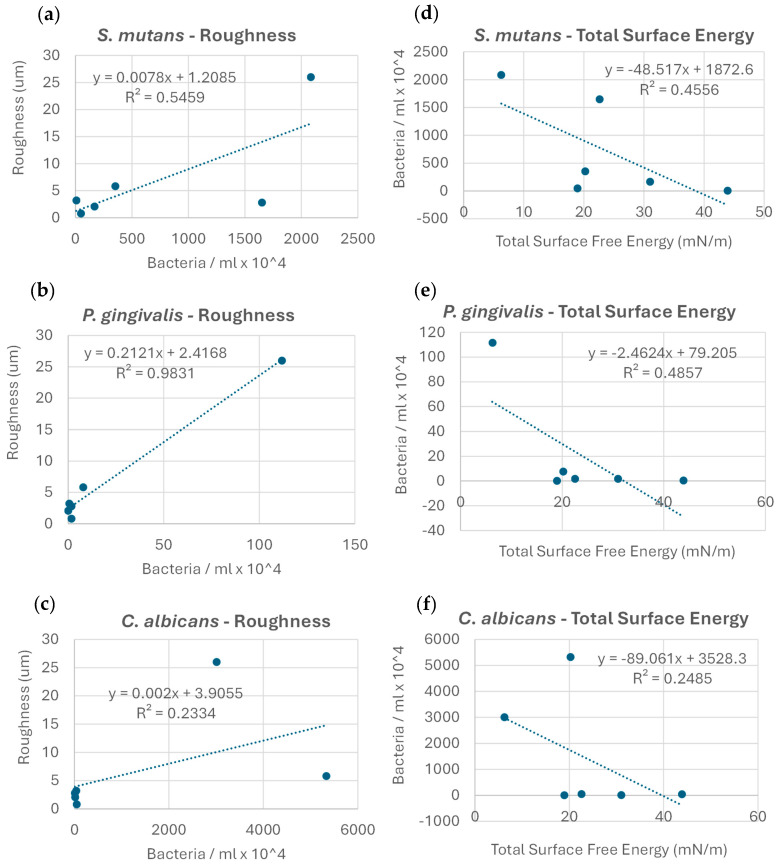
Correlations between the extent of microbial adhesion, membrane surface roughness (**a**–**c**), and total surface energy (**d**–**f**) for the microorganisms examined.

No clear correlation was found between the microbial adhesion extent and Gibbs free energy change values (r^2^ = 0.05). Upon examining the exact correlation between the extent of microbial adhesion and the membrane roughness values for each strain individually, a clear trend in microbial adhesion increase with increases in roughness was observed; the correlation was the most robust for *P. gingivalis* (r^2^ = 0.98), followed by *S. mutans* (r^2^ = 0.55), and less so for *C. albicans* (r^2^ = 0.23) (Figure 4a–c). The correlation between the microbial adhesion extent and membrane total SFE within strains showed a clear decreasing trend in microbial adhesion, with an increase in the total SFE; this correlation was strongest for *P. gingivalis* (r^2^ = 0.49), followed by *S. mutans* (r^2^ = 0.46), and less so for *C. albicans* (r^2^ = 0.25) (Figure 4d–f).

## 4. Discussion

In this study, the SFE and roughness of six commercially available PTFE membranes used in GBR were evaluated. In addition, microbial adhesion to the membrane surfaces was investigated, as it is a critical step that initiates biofilm growth, which may subsequently lead to infections [20]. The polar SFE of all six membranes was very low, indicating surface non-polarity [21,22].

The PTFE membranes were hydrophobic, with the CA of water being larger than that of DII, indicating differences in surface polarity. The wetting of a solid surface by a liquid depends not only on chemistry, but also on surface morphology [23]. Reflecting this, no difference between fluids was observed with the Surgitime membrane due to its surface polarity and roughness, suggesting that its surface was hydro- and oleophobic. For comparison, the OsseoGuard^®^-TXT and Cytoplast^TM^ 200-TXT membranes had hexagonal indentations and much less surface roughness than the Surgitime membrane. The decreased wettability of these membranes follows the Wenzel model, with additional roughness increasing the surface hydrophobicity of materials such as PTFE [24]. The Surgitime membrane also had the least total SFE, which, combined with its surface roughness, enhanced microbial adhesion and growth, consistent with previous findings [25,26]. Wettability and roughness were positively correlated for all six membranes (i.e., roughness enhanced hydrophobicity), given that the influence of the surface roughness on wettability is stronger than that of the SFE, especially for hydrophobic surfaces and hydrophilic liquids such as water [27,28]. Some studies suggest that the correlation between roughness and microbial adhesion is the most critical factor, whereas others suggest that hydrophobicity is paramount [27,28,29]. The differences in the SFE among membranes in this study might have been due to differences in the surface architecture or surface treatment, which affected the membranes’ crystallinity and wettability [25].

The biomaterial surface hydrophobicity and roughness are known to affect microbial adhesion and subsequent biofilm formation and infection [30], and all the microbes tested adhered to the six PTFE membranes in this study. These results align with previous findings [3] and are congruent with thermodynamic theory and the negative Gibbs free energy values obtained in this study, which showed that hydrophobicity is thermodynamically favorable [18]. Although no correlation between the extent of microbial adhesion and Gibbs free energy changes was demonstrated, thermodynamic theory predicted the microbial adhesion observed.

Similarly to material hydrophobicity, microorganism hydrophobicity increases adhesion [31]. Furthermore, oral microorganisms, which typically have a high SFE, adhere better to high-energy solids and are unlikely to adhere to substrates with a low SFE [13,31,32]. Our bacterial hydrophobicity results confirm these patterns; *S. mutans* was the most hydrophobic and had the least polar SFE among the microorganisms. The weak correlation for *C. albicans* suggests that its unique dimorphic growth behavior, biofilm-forming ability, and broader range of adhesion factors may account for its variable interaction with PTFE surfaces compared to bacterial species. An investigation by De-la-pinta et al. [30] reported that while the material roughness did affect microbial adhesion and biofilm formation, hydrophobicity had a more pronounced influence, often overshadowing roughness effects. Specifically, highly hydrophobic materials (such as Teflon and silicone) allowed for greater biofilm formation by *C. albicans*, regardless of the surface roughness differences. Conversely, surfaces with intermediate roughness but higher hydrophilicity (e.g., titanium) showed significantly lower *C. albicans* biofilm formation, indicating that hydrophobicity could mitigate or amplify the impact of surface roughness. Therefore, our weak correlation aligns with these observations, emphasizing the finding that biofilm formation by *C. albicans* on GBR membranes is likely more significantly influenced by hydrophobic interactions than roughness alone.

In this study, the Surgitime membrane (with a low SFE and high surface roughness) attracted the most *S. mutans* and *P. gingivalis* and the second most *C. albicans*. All three species adhered less to the OsseoGard^®^-TXT, OsseoGuard^®^-NTXT, and permamem^®^ membranes due to their high SFE and low surface roughness. The Cytoplast^TM^ TXT-200 membrane attracted the most *C. albicans*, which can be partly explained by its high surface roughness and low SFE. The NeoGen^®^ membrane attracted the second most *S. mutans*, but fewer *P. gingivalis* and *C. albicans*, which can be partly explained by its moderate roughness, low total SFE, and non-polarity.

Recent studies have highlighted how the membrane microstructure and composition critically influence bacterial adhesion in guided bone/tissue regeneration (GBR/GTR). The crystallinity and surface topography of d-PTFE membranes significantly affect microbial adhesion, with higher crystallinity (78.6% vs. 34.2%) increasing adhesion by ~1 log_10_CFU/mL for oral bacteria such as *S. mutans* and *A. actinomycetemcomitans* [25]. Comparative analyses of commercial membranes (e.g., Lumina PTFE^®^ vs. collagen-based Bio-Gide^®^) have revealed that PTFE membranes better resist adhesion [33]. Earlier studies onadhesion further demonstrate that collagen membranes attract stronger colonization by *S. mutans* and *P. gingivalis* than ePTFE or polyglactin 910 [3], as corroborated by findings indicating that collagen promotes higher adherence of periodontal pathogens such as *P. gingivalis* compared to PTFE [34]. Taken together, these studies underscore the need to optimize the physicochemical properties of membranes to mitigate infection risks in regenerative dentistry.

A limitation of this study is the absence of in vivo validation. Microbial adhesion was assessed under controlled laboratory conditions, which may not fully represent the complex and dynamic oral environment, given that the oral cavity hosts diverse microbial species, and their interactions can influence bacterial adhesion. Additionally, membranes are exposed to salivary proteins, which may modify surface roughness and wettability, affecting microbial adhesion. Additionally, surface modifications such as sintering can change membrane properties—including crystallinity, stiffness, and potentially surface charge—which may significantly affect microbial adhesion patterns [25,35,36]. The responses of different microorganisms to such material parameters are known to differ [25,37,38]. Investigating these factors in future research could yield more clinically reliable results.

## 5. Conclusions

The findings of this study suggest that the performance of GBR-PTFE membranes in terms of microbial adhesion depends on the surface roughness and SFE of the membrane and the hydrophobicity of the microorganism as well. *P. gingivalis*, the most hydrophilic microbe, adhered the least to all tested membranes. Concurrently, we observed that Surgitime, characterized by the highest surface roughness and the lowest surface free energy (SFE), exhibited the greatest microbial adhesion for *S. mutans* and *P. gingivalis*, whereas *C. albicans* adhered most prominently to Cytoplast™ TXT-200. By contrast, membranes with a lower surface roughness and higher SFE, such as OsseoGuard-TXT, consistently showed the least microbial adhesion across all the tested species. These findings suggest that both surface topography and energy significantly influence microbial colonization patterns and may offer valuable guidance in the clinical selection of PTFE membranes, particularly in scenarios where membrane exposure is anticipated during guided bone regeneration procedures.

## Figures and Tables

**Table 1:** Specifications of the membranes examined.

Membranes	Structure	Surface	Manufacturer
permamem^®^	hd-PTFE	NTXT	Botiss biomaterials GmbH, Zossen, Germany
Cytoplast^TM^ TXT-200	hd-PTFE	TXT	Osteogenics Biomedical Inc., Lubbox, TX, USA
NeoGen^®^	Dual e-PTFE	Tight/expanded texture NTXT	Neoss Ltd., Harrogate, UK
OsseoGuard^®^-TXT	hd-PTFE	TXT	ZimVie, PB Gardens, FL, USA
OsseoGuard^®^-NTXT	hd-PTFE	NTXT	ZimVie, PB Gardens, FL, USA
Surgitime	PTFE	NTXT	Bionnovation Biomedical, São Paulo, Brazil

hd: high-density PTFE membranes, commonly known as dPTFE, characterized by minimal porosity and non-permeability to bacteria. TXT: indicates a textured surface, referring to membranes that have intentional surface micro- or macro-patterning (hexagonal-shaped indentations) to aid mechanical handling or soft tissue integration. NTXT: indicates a non-textured (smooth) surface, referring to membranes with minimal surface relief and lower surface roughness.

**Table 2:** Roughness, SFE, and CA of the examined PTFE membranes and the microbial strains (mean ± SD).

	Roughness (Ra) (µm)	Water CA (°)	DII CA (°)	Total SFE (mN/m)	Dispersive SFE (mN/m)	Polar SFE (mN/m)
Cytoplast^TM^ TXT-200	5.82 ± 1.34	96.87 ± 0.6	77.81 ± 0.33	19 ± 0.10	18.38 ± 0.10	1.08 ± 0.01
OsseoGuard^®^-TXT	3.20 ± 1.10	66.31 ± 9.15	54.84 ± 0.86	39.87 ± 1.42	31.89 ± 0.47	8.34 ± 1.29
permamem^®^	2.0813 ± 0.4	119.13 ± 3.89	57.44 ± 0.37	30 ± 3.92	29.67 ± 3.97	0.62 ± 0.05
Surgitime	24.1 ± 6.9	118.56 ± 0.06	113.28 ± 0.69	5.61 ± 0.12	4.53 ± 0.16	1.12 ± 0.04
OsseoGuard^®^-NTXT	0.80 ± 0.12	111.59 ± 0.30	77.53 ± 0.29	18 ± 0.15	18.54 ± 0.15	0.11 ± 0.01
NeoGen^®^	2.80 ± 0.7	107.81 ± 0.24	70.52 ± 2.17	22.87 ± 1.11	23.17 ± 1.14	0.00 ± 0.03
*S. mutans*	---	27.6 ± 2.68	39.98 ± 0.74	69.87 ± 1.94	39.63 ± 0.53	30.23 ± 0.53
*P. gingivalis*	---	18.35 ± 3.3	49.06 ± 0.68	71.75 ± 1.76	34.81 ± 0.53	36.94 ± 1.23
*C. albicans*	---	22.11 ± 1.83	55.09 ± 1.04	69.30 ± 1.30	31.41 ± 0.84	37.89 ± 0.46

CA: contact angle; DII: diiodomethane; and SFE: surface free energy.

## Data Availability

The processed data required to reproduce these findings cannot be shared at this time, as the data are currently utilized in ongoing research.

## Supplementary Materials

The following supporting information can be downloaded at: https://www.mdpi.com/article/10.3390/dj13070301/s1, Figure S1: SEM image of a PTFE membrane. The arrows indicate representative flat areas between the hexagonal-shaped indentations selected for measurements; Table S1: Species-specific primers used for 16S rRNA genes.
