# Supplementary material for: In Vitro Microbial Adhesion on the Surfaces of Various Polytetrafluoroethylene Membranes Used in Guided Bone Regeneration
Source: Dent J (Basel). 2025 Jul 2;13(7):301. doi: 10.3390/dj13070301 (PMC12293225; doi:10.3390/dj13070301)
Supplement: Supplementary file 1 [file dentistry-13-00301-s001.zip › dentistry-3657918-supplementary.pdf]

Supplementary Data

In vitro Microbial Adhesion on the Surface of Various PTFE Membranes used in Guided Bone Regeneration

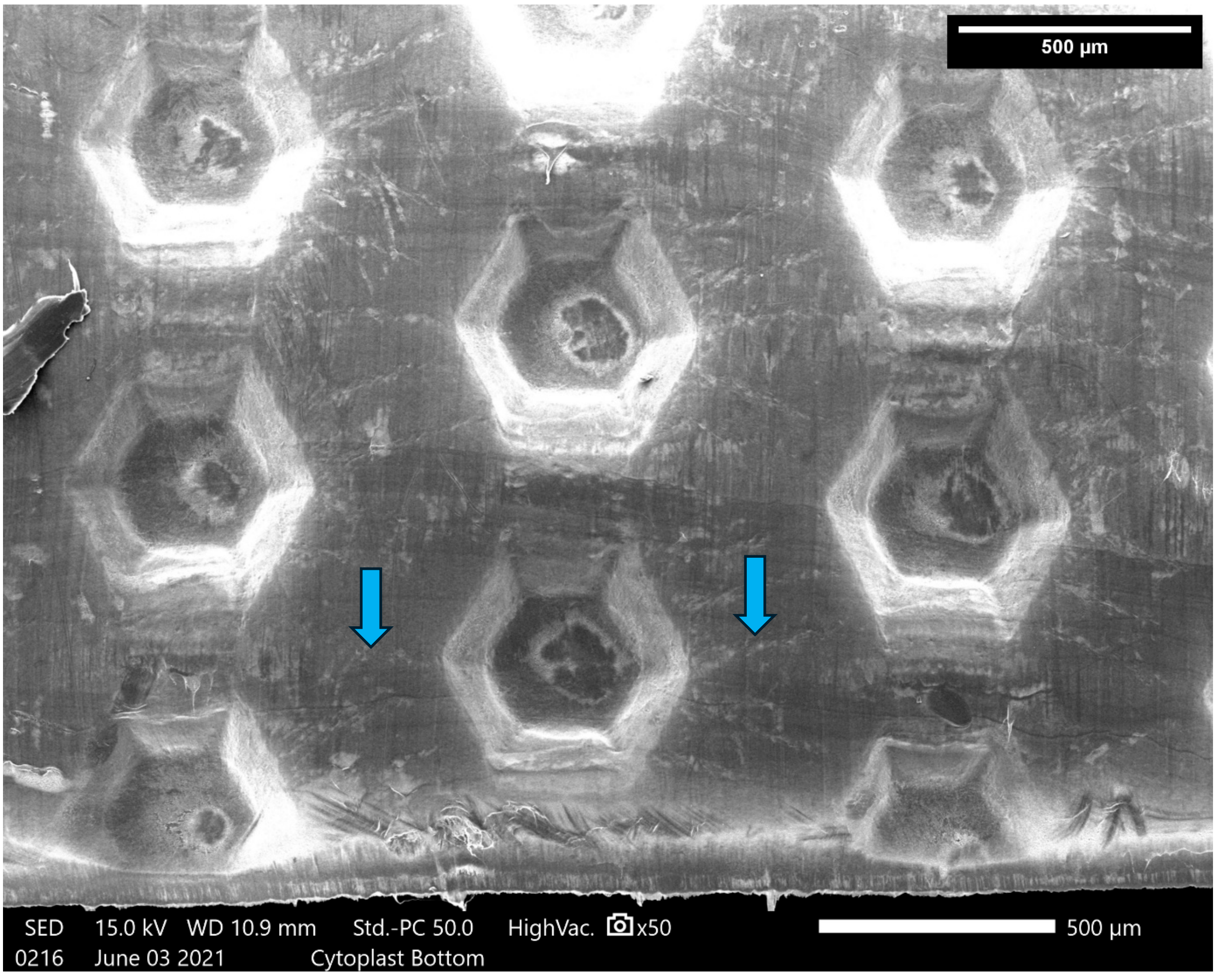

Suppl. Figure S1. SEM image of a PTFE membrane. The arrows indicate representative flat areas between the hexagonal-shaped indentations selected for measurements.

Suppl. Table S1. Species-specific primers used for 16S rRNA genes.

Microorganisms	Forward	Reverse	References
S. mutans	TCGCGAAAAAGATAAACAAACA	GCCCCTTCACAGTTGGTTAG	16
P. gingivalis	AGGCAGCTTGCCATACTGCG	ACTGTTAGCAACTACCGATGT	16
C. albicans	TCAACTTGTCACACCAGATTATT	TCC TCC GCT TAT TGA TAT GC	17

Contact Angle (CA) measurements according to the method by Owens & Wendt, Rabel and Kaelble (OWRK).

The CA measurements of the two liquids allowed the calculations of the polar and dispersive components of substrates and microorganisms SFE, using the following equation:

$$1+\cos\theta = 2\sqrt{\gamma_S^d}\left(\frac{\sqrt{\gamma_L^d}}{\gamma_L}\right)+2\sqrt{\gamma_S^p}\left(\frac{\sqrt{\gamma_L^p}}{\gamma_L}\right)$$

The dispersion-polar approach of the Thermodynamic Theory.

According to this approach, the free energy of adhesion of a bacterium (B) to a substratum surface (S) in a suspending liquid (L), is expressed as the sum of dispersion (d) and polar (p) adhesion energies according to the equation:

$$\Delta G_{adh}^{d-p} = \Delta G_{d0}^{d} + \Delta G_{d0}^{p} =$$

$$= \left[\left(\sqrt{\gamma_B^d}-\sqrt{\gamma_S^d}\right)^2 - \left(\sqrt{\gamma_B^d}-\sqrt{\gamma_L^d}\right)^2 - \left(\sqrt{\gamma_S^d}-\sqrt{\gamma_L^d}\right)^2\right] + \left[\left(\sqrt{\gamma_B^p}-\sqrt{\gamma_S^p}\right)^2 - \left(\sqrt{\gamma_B^p}-\sqrt{\gamma_L^p}\right)^2 - \left(\sqrt{\gamma_S^p}-\sqrt{\gamma_L^p}\right)^2\right]$$

ΔG_{adh}^{d-p} is the total free energy of adhesion, and ΔG_{d0}^{d}, ΔG_{d0}^{p} is its dispersion and polar component respectively. γ_{BS} (J/m^2) is the bacteria-substrate interfacial free energy, γ_{BL} (J/m^2) is the bacteria-liquid interfacial free energy and γ_{SL} (J/m^2) is the substrate-liquid interfacial free energy. In order to evaluate the Gibbs free energy changes (ΔG_{adh}^{d-p}) upon adhesion of a bacterium (B) to a substratum surface (S) in a suspending liquid (L), according to the dispersion-polar approach, it is therefore necessary to calculate the dispersion (γ_S^d) and the polar (γ_S^p) components of the surface free energy for the material and the bacterial surfaces,

since for the water -and other liquids- γ_L, γ_L^d and γ_L^p are quite well known. Adhesion is favored if $\Delta G^{adh} < 0$, which means that spontaneous attachment is accompanied by a decrease in the free energy of the system.

References

16. Karched M, Bhardwaj RG, Inbamani A, Asikainen S. Quantitation of biofilm and planktonic life forms of coexisting periodontal species. *Anaerobe*. **2015**;35(Pt A):13-20.

17. Li YL, Leaw SN, Chen JH, Chang HC, Chang TC. Rapid identification of yeasts commonly found in positive blood cultures by amplification of the internal transcribed spacer regions 1 and 2. *Eur J Clin Microbiol Infect Dis*. **2003**;22(11):693-6.
